# Identifying alcohol problems among suicide attempters visiting the emergency department

**DOI:** 10.1186/s12888-019-2347-5

**Published:** 2019-11-08

**Authors:** Jinhee Lee, Seongho Min, Joung-Sook Ahn, Hyun Kim, Yong-Sung Cha, Eugene Oh, Jin Sil Moon, Min-Hyuk Kim

**Affiliations:** 1 Department of Psychiatry, Yonsei University Wonju College of Medicine, 20 Ilsan-ro, Wonju, 26426 Republic of Korea; 2 Department of Emergency Medicine, Yonsei University Wonju College of Medicine, Wonju, Republic of Korea; 3 Yonsei-Naru Psychiatric Clinic, Seoul, Republic of Korea; 4 Center of Biomedical Data Science, Yonsei University Wonju College of Medicine, Wonju, South Korea

**Keywords:** Suicide, Alcohol, Emergency department

## Abstract

**Background:**

Many suicide attempters brought to our emergency department (ED) have been found to have alcohol problems, and this should be taken serious consideration because alcohol use disorder is a risk factor for suicide reattempt. In this study, we aimed to estimate the effectiveness of alcohol-related biochemical markers and Alcohol Use Disorder Identification Test Consumption (AUDIT-C) in suicide attempters who visited our ED based on the gold standard for clinical diagnosis used by psychiatrists for alcohol use disorder. Moreover, we aimed to search for a significant standard when clinicians make correct predictions about alcohol use disorder using these markers.

**Methods:**

Among the subjects who visited ED following a suicide attempt, a total of 203 subjects were selected. Following a psychiatric interview, the subjects who met the criteria for alcohol abuse or alcohol dependence according to DSM-IV-TR in the past year were defined as the “alcohol use disorder” group. Although some subjects did not meet these criteria, men with a weekly alcohol intake of ≥14 drinks and women with a weekly alcohol intake of ≥7 drinks were classified as the “risky drinking” group. AUDIT-C was used as a self-report; further, aspartate aminotransferase, gamma-glutamyltransferase (GGT), and carbohydrate-deficient transferrin (CDT) were assayed using standard methods, and GGT–CDT was calculated using this formula: 0.8 × ln(GGT) + 1.3 × ln(%CDT).

**Results:**

In total, 88 subjects met the criteria for alcohol use disorder and 115 were included in the reference group. In the screening for alcohol use disorder, the AUC of AUDIT-C was 0.89 for men and 0.87 for women. In the screening for risky drinking, the AUC of AUDIT-C was 0.99 for men and 0.93 for women. Compared with other biochemical markers, AUDIT-C showed the highest AUC value for screening for both alcohol use disorder and risky drinking, with the trend being more prominent in men.

**Conclusions:**

Among the biochemical markers, AUDIT-C yielded the highest sensitivity, specificity, and accuracy in diagnosing alcohol use disorder among suicide attempters in ED. Comparison of results revealed that the use of AUDIT-C with biochemical markers or its use alone can help screen for alcohol use disorder or risky drinking in clinical settings.

## Background

According to the World Health Organization report, approximately 1 million people die by suicide annually. In Korea, the suicide rate per 100,000 population was 25.8 in 2017, which was approximately 2.2 times higher than that reported by the Organisation for Economic Co-operation and Development (average: 11.6 deaths per 100,000 people) [1]. Suicidal signs are known to occur because of the combined effects of various factors, but numerous studies have reported their particular relation with alcoholism [2, 3]. In Korea, which has a high suicide rate, the per capita consumption of alcohol is 7.95 L and the annual drinking rate among adults is approximately 77%, which is far above the global average reported by the WHO statistics in 2014. This unusually high alcohol consumption rate suggests its strong association with the high suicide rate in Korea [4].

Alcohol is consumed widely worldwide and is known to cause not only various physical diseases but also impulse control disorders such as violence, self-harm, and suicide. Suicide attempters are commonly found in an acute alcoholic state [5]; in previous studies, 33–66% of suicide victims were reported to have positive findings for blood alcohol test [6, 7]. In addition, patients with alcohol dependence are more likely to commit impulsive and fatal attempts, [8] and alcohol itself is known as an important risk factor for suicide reattempt [9–11]. Therefore, the early detection of drinking problems and early interventions for suicide attempters who visit the emergency department (ED) are important to reduce the risk of suicide reattempt. However, among suicide attempters, obtaining the exact related history is often difficult because of mental confusion or physical problems, and they tend to under-report or hide their problems [12, 13]. Difficulties in detecting alcohol problems among suicide attempters in emergency situations include limited time, manpower, and treatment based only on acute symptoms.

A formal self-report questionnaire such as Alcohol Use Disorder Identification Test Consumption (AUDIT-C) is the most commonly recommended screening tool for diagnosing alcohol use disorder [14, 15]. Biochemical markers such as gamma-glutamyltransferase (GGT), mean corpuscular volume (MCV), and percent carbohydrate-deficient transferrin (%CDT), or transferrin isoforms, have also been used to detect patients with heavy alcohol consumption [16–18]. Numerous studies have compared the efficacy of screening tools and biomarkers [19–21] in ED. A study on trauma patients who visited ED found that the screening properties of AUDIT are superior to those of %CDT, MCV, and GGT for the diagnosis of alcohol use disorder [19]. In contrast, another study on patients undergoing emergency general surgery reported that AUDIT was neither objective nor had the sensitivity to identify patients practicing heavy alcohol consumption [21]. Previous studies have not reached consistent conclusions, and limited research has been conducted on the discrimination of alcohol problems among suicide attempters in ED. Therefore, in this study, we aimed to evaluate the diagnostic accuracy of AUDIT-C and alcohol-related biochemical markers in identifying individuals with risky drinking and alcohol use disorder among suicide attempters who visited our ED.

## Methods

### Subjects

**Fig. 1:**
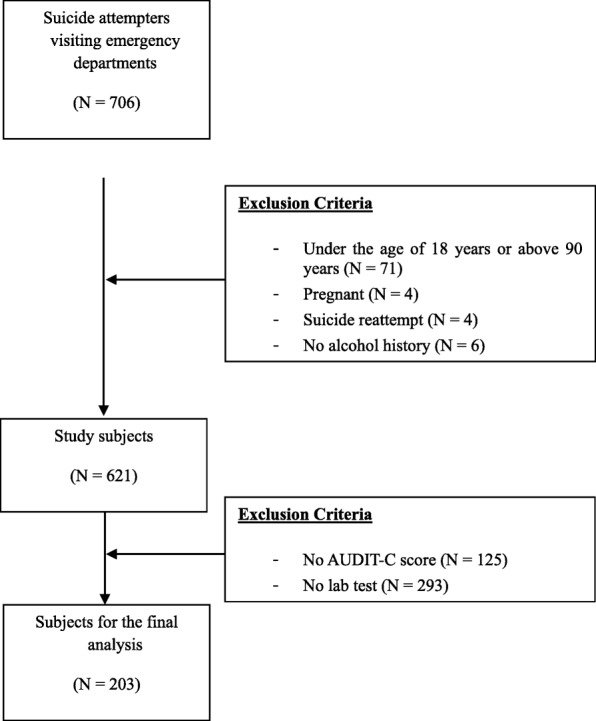
Flowchart of the study design

Among the subjects who visited the Emergency Room of the Wonju Severance Christian Hospital following a suicide attempt between October 2010 and August 2013, a total of 706 suicide attempters were approached for inclusion in the current study. We defined “suicide attempts” as incidents when subjects and/or their caregivers reported an obvious intent. Only when the suicide attempters were unable to provide reliable information due to mental confusion or severe physical damages, medical staff confirmed the injuries as a result of a possible suicide attempt based on clear evidences (i.e., obvious circumstance and witness report) [22]. All subjects agreed for a psychiatric interview and blood collection to test for biochemical markers after they were completely informed about the aims and methods of the study. In total, 71 suicide attempters aged < 18 or > 90 years at the time of their hospital visit were excluded. Four suicide attempters who were pregnant and four suicide reattempters were also excluded because only those who came for the first time were included. Six suicide attempters without any history of alcohol use because they were unavailable for the evaluation of the history of alcohol use or had refused to report such a history were excluded. Among 621 subjects, the data of 203 suicide attempters were finally analyzed in the study, excluding the data of those who did not have an AUDIT-C score and laboratory findings (Fig. 1).

### Psychiatric interview and assessment of alcohol use disorder and risky drinking

After the evaluation of the physical condition of the subjects by the ED physician, a psychiatric interview was conducted with the subjects and their family members to evaluate the alcohol use problem and psychiatric disorder. If a patient had difficulty in communicating due to poor consciousness, his/her information was collected through the caregiver and/or through revisit and reinterview after the patient regained consciousness. A psychiatric interview was conducted for all subjects, which included questions on sociodemographic variables such as age, sex, educational level, marital status, and smoking status. Moreover, the method of suicide attempt (drug intoxication/hanging/wrist cutting/CO intoxication/others) was assessed through the psychiatric interview for all subjects. The medical record of each patient diagnosed using DSM-IV-TR [23] based on the diagnostic consensus of two psychiatrists who did not know the subjects’ AUDIT-C scores was reviewed; then, the alcohol use disorder group was defined. In addition, according to the definition of NIAAA, the risky drinking group was composed of patients with an average weekly alcohol intake of > 14 glasses among men and > 7 glasses among women [24]. The control group was composed of subjects who were not included in the risky drinking or nondrinking groups.

### Audit-c

AUDIT-C, which comprises the first 3 items among the 10 items of AUDIT, is scored between 0 and 12 points. It is an effective, brief alcohol screening questionnaire for risky drinking or alcohol use disorder [25]. The validity of AUDIT-C has been proven in previous studies. In a study on the data of veterans in the United States, > 4 points were allocated to men and 3 to women in AUDIT-C, which were confirmed as the cutoff points for risky drinking, [14, 26] and the same results were observed in another study conducted on the general population [25]. In some European studies, > 5 points were defined as the cutoff point [18, 27, 28] for alcohol use disorder. Some previous studies conducted in Korea recommend a cutoff point higher than those recommended in studies conducted in other countries such as > 6 points in men and 4 [29] or 8 points [30] in women. In the present study, we assessed 3 items of AUDIT-C through interviews with suicide attempters at the time of visit to our ED, and the cutoff point was determined by ROC analysis.

### Biochemical markers

We collected blood samples of suicide attempters who agreed to participate in the study and measured the levels of biochemical markers known to be related to alcohol use disorder.

AST, which is used widely to detect alcohol-induced liver disease, was measured using MODULAR analyzers from Roche/Hitachi. The sensitivity and specificity of AST for liver damage due to alcohol use vary from 10 to 90% depending on the study, [18] but it is used widely because it is economical and easy to measure. AST has been known to have approximately 90% sensitivity to monitor the recurrence of alcohol use disorder, but it has some limits to clinical use because of its low specificity [31].

GGT was also measured using MODULAR analyzers from Roche/Hitachi. An increased serum GGT level in subjects with alcohol use disorder is due to their response to increased oxidative stress in liver injury and consequent reduction in GGT levels. Binge drinking may increase serum GGT levels due to hepatic necrosis [31]. The sensitivity of GGT to alcohol differs among studies, but it is usually higher than those of other alcohol-related biochemical markers [32–34].

We used Capillarys 2 by SEBIA using capillary zone electrophoresis to measure %CDT. %CDT is known to be the most specific marker for chronic alcohol consumption to date and the only experimental marker to be certified by the US Food and Drug Administration [35, 36].

A number of studies have indicated higher sensitivities for the combined use of GGT and CDT than for the use of GGT and CDT alone to detect alcohol use disorder, [37–39] and a strong association has been reported between these markers and the volume of drinking [37]. In this context, we also calculated the GGT–CDT value using the formula proposed in previous studies: 0.8 × ln(GGT) + 1.3 × ln(%CDT) [40].

### Statistical analysis

Descriptive statistics were obtained to confirm the basic characteristics of the subjects. Chi-square test was used for categorical data and independent *t*-test for continuous data. Men and women with significant differences were stratified. The effects of AUDIT-C and each biochemical biomarker on alcohol use disorder and risky drinking were examined. Multivariate logistic regression analysis was performed after adjusting for age, marital status, educational level, and smoking status [41].

Receiver operating characteristic (ROC) curves were plotted for each prediction probability value obtained from the multivariate logistic regression. The optimal prognostic factors were selected by plotting the ROC curve using each prediction probability value obtained from the multivariate logistic regression analysis and by comparing the sensitivity, specificity, likelihood ratio, and accuracy statistics of each biomarker. SAS 9.4 and MedCalc were used as statistical analysis tools, and statistical significance was set at a *p*-value of < 0.05.

## Results

A total of 203 subjects (96 men and 107 women) were assessed in this study. Table 1 presents the demographic characteristics of the study subjects according to sex. The prevalence of alcohol use disorder and risky drinking was significantly higher among men than among women (*p* < 0.001). Among the subjects, 58 men (60.4%) and 30 women (28.0%) were diagnosed with alcohol use disorder and 65 men (67.8%) and 49 women (45.8%) met the criteria for risky drinking. A higher number of men were current smokers or ex-smokers than that of women (*p* < 0.001). However, no significant sex differences were found in the method of suicide attempt, with the most common method being drug intoxication.

**Table 1:** Demographic characteristics of the study subjects

	Men	Women	χ^2^/t	*p*-value
	(*N* = 96)	(*N* = 107)		
Mean age (SD)	49.25 (14.776)	45.92 (17.086)	2.188	0.141
Alcohol use disorder (%)				
Reference	38 (39.6%)	77 (72.0%)	21.603	0.0001
Alcohol use disorder^*^	58 (60.4%)	30 (28.0%)		
Risky drinking (%)				
Reference	31 (32.2%)	58 (54.2%)	11.899	0.001
Risky drink^**^	65 (67.8%)	49 (45.8%)		
Suicide attempt method (%)				
Dug intoxication	66 (86.8%)	89 (83.2%)	8.793	0.059
Hanging	8 (8.3%)	3 (2.8%)		
Wrist cutting	6 (6.3%)	8 (7.5%)		
CO intoxication	12 (12.5%)	5 (4.7%)		
Others	4 (4.2%)	2 (1.9%)		
Smoking (%)				
Never smoker	20 (21.1%)	67 (62.6%)	35.678	0.0001
Current smoker	61 (64.2%)	34 (31.8%)		
Cessation	14 (14.7%)	6 (5.6%)		
Marital status (%)				
Single or unmarried	22 (22.9%)	18 (16.8)	1.4034	0.4957
Separated/Divorced/Widowed	16 (16.7%)	22 (20.6)		
Married/Cohabitation	58 (60.4%)	67 (62.6)		
Educational level (%)				
None	10 (10.4%)	11 (10.3%)	2.1582	0.3399
Elementary/Middle school	47 (49.0%)	42 (39.2%)		
High school/College	39 (40.6%)	54 (50.5%)		

^*^Alcohol use disorder (alcohol abuse or alcohol dependence in DSM-IV-TR)/reference population (not meet the substance use disorder criteria in DSM-IV-TR)

^**^Risky drinking (men > 14 drinks/week, women > 7 drinks/week)/reference population (men ≤14 drinks/week, women ≤7 drinks/week)

**Fig. 2:**
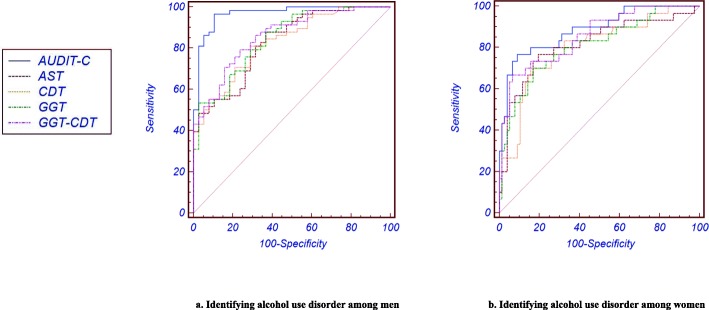
Receiver operating characteristic curves of AUDIT-C and biochemical markers for alcohol use disorder. a. Identifying alcohol use disorder among men. b. Identifying alcohol use disorder among women

Among the subjects with alcohol use disorder, AUDIT-C revealed an AUC of 0.97 for men and 0.89 for women, which was a stand-alone instrument that performed significantly better than other biochemical markers (AST: 0.83, GGT: 0.85, CDT: 0.83, and GGT–CDT: 0.86 in men; AST: 0.82, GGT: 0.81, CDT: 0.80, GGT–CDT: 0.86 in women) (Fig. 2). AUDIT-C also showed the highest sensitivities among men (96.5) and women (76.7). The specificity of AUDIT-C was the highest among men (89.5); however, the specificity of GGT–CDT (93.5) was higher than AUDIT-C (90.9) among women (Table 2).

**Table 2:** Performance of AUDIT-C and biochemical markers for detecting alcohol use disorder

	Alcohol use disorder^*****^											
	Men (*N* = 58)						Women (*N* = 30)					
	AUC (95% CI)	SEN (95% CI)	SPC (95% CI)	LR+	LR−	Accuracy (95% CI)	AUC (95% CI)	SEN (95% CI)	SPC (95% CI)	LR+	LR−	Accuracy (95% CI)
AST^*^	0.83 (0.74; 0.90)	87.9 (76.7; 95.0)	63.2 (46.0; 78.2)	2.39	0.19	0.77 (0.67; 0.85)	0.82 (0.73; 0.88)	76.7 (87.7; 90.1)	80.5 (69.9; 88.7)	3.94	0.29	0.79 (0.70; 0.86)
GGT^*^	0.85 (0.76; 0.91)	87.9 (76.7; 95.0)	63.2 (46.0; 78.2)	2.39	0.19	0.77 (0.67; 0.85)	0.81 (0.72; 0.88)	70.0 (50.6; 85.3)	83.1 (72.9; 90.7)	4.15	0.36	0.79 (0.71; 0.87)
CDT^**^	0.83 (0.74; 0.90)	81.0 (68.6; 90.1)	71.1 (54.1; 84.6)	2.80	0.27	0.76 (0.66; 0.84)	0.80 (0.71; 0.87)	70.0 (50.6; 85.3)	84.4 (74.4; 91.7)	4.49	0.36	0.79 (0.71; 0.87)
GGT–CDT^***^	0.86 (0.77; 0.92)	79.3 (66.6; 88.8)	76.3 (59.8; 88.6)	3.35	0.27	0.77 (0.67; 0.85)	0.86 (0.77; 0.92)	66.7 (47.2; 82.7)	93.5 (85.5; 97.9)	10.27	0.36	0.79 (0.71; 0.87)
AUDIT-C^****^	0.97 (0.91;0.99)	96.5 (88.1;99.6)	89.5 (75.2;97.1)	9.17	0.04	0.94 (0.87;0.98)	0.89 (0.81;0.94)	76.7 (57.7;90.1)	90.9 (82.2;96.3)	8.43	0.26	0.87 (0.79;0.93)

^*^Cutoff points for AST and GGT are 40 U/L

^**^Cutoff point for CDT – “all” group is 1.0%, “men” group is 1.0%, and “women” group is 0.8%

^***^Cutoff point for GGT–CDT – “all” group is 2.9308, “men” group is 3.0737, and “women” group is 2.2183

^****^Cutoff point for AUDIT-C – “all” group is score 4, “men” group is score 4, and “women” group is score 8. (The cutoff point was determined by ROC analyses in the sample)

^*****^Alcohol use disorder (alcohol abuse or alcohol dependence in DSM-IV-TR)/reference population (not meet the substance use disorder criteria in DSM-IV-TR)

AST = aspartic acid transaminase; AUC = area under the curve; AUDIT-C = Alcohol Use Disorder Identification Test Consumption; CDT = carbohydrate-deficient transferrin; GGT = gamma-glutamyltransferase; LR = likelihood ratios; SEN = sensitivity; SPC = specificity

**Fig. 3:**
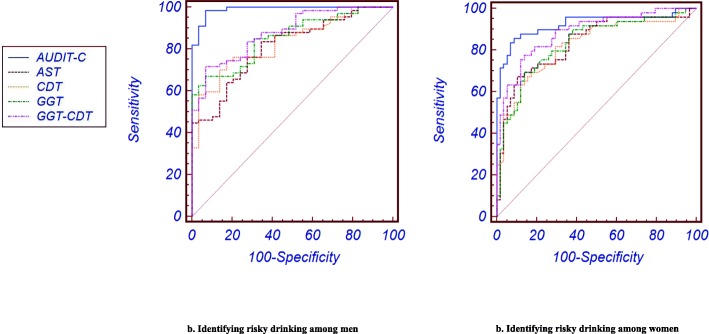
Receiver operating characteristic curves of AUDIT-C and biochemical markers for risky drinking. a. Identifying risky drinking among men. b. Identifying risky drinking among women

Among the subjects with risky drinking, AUDIT-C revealed a higher AUC value than other biochemical markers (AST: 0.81, GGT: 0.85, CDT: 0.83, and GGT–CDT: 0.88 in men; AST: 0.84, GGT: 0.83, CDT: 0.82, and GGT–CDT: 0.89 in women) among men (0.99) and women (0.93) (Fig. 3). The sensitivity of AUDIT-C in this group was also the highest among men (98.5) and women (85.7). Notably, the specificity of AUDIT-C (93.7) was the same as those of GGT (93.7) and GGT–CDT (93.7) among men, but among women, AUDIT-C (91.4) showed a higher specificity than other biochemical markers (Table 3).

**Table 3:** Performance of AUDIT-C and biochemical markers for detecting risky drinking

	Risky drinking^*****^											
	Men (*N* = 65)						Women (*N* = 49)					
	AUC (95% CI)	SEN (95% CI)	SPC (95% CI)	LR+	LR−	Accuracy (95% CI)	AUC (95% CI)	SEN (95% CI)	SPC (95% CI)	LR+	LR−	Accuracy (95% CI)
AST^*^	0.81 (0.72;0.88)	83.6 (72.5;91.5)	65.5 (45.7;82.1)	2.42	0.25	0.76 (0.66;0.84)	0.84 (0.75;0.90)	67.4 (52.5;80.1)	89.7 (78.8;96.1)	6.51	0.36	0.74 (0.64;0.82)
GGT^*^	0.85 (0.77;0.92)	67.2 (54.6;78.2)	93.1 (77.2;99.2)	9.74	0.35	0.76 (0.66;0.84)	0.83 (0.75;0.90)	69.4 (54.6;81.7)	86.2 (74.6;93.9)	5.03	0.36	0.73 (0.63;0.81)
CDT^**^	0.83 (0.74;0.90)	74.6 (62.5;84.5)	82.8 (64.2;94.2)	4.33	0.31	0.73 (0.63;0.81)	0.82 (0.74;0.89)	81.6 (68.0;91.2)	70.7 (57.3;81.9)	2.79	0.26	0.73 (0.63;0.81)
GGT–CDT^***^	0.88 (0.80;0.94)	71.6 (59.3;82.0)	93.1 (77.2;99.2)	10.39	0.30	0.76 (0.66;0.84)	0.89 (0.81;0.94)	77.6 (63.4;88.2)	86.2 (74.6;93.9)	5.62	0.26	0.75 (0.65;0.83)
AUDIT-C^****^	0.99 (0.94;1.00)	98.5 (92.0;100.0)	93.1 (77.2;99.2)	14.28	0.02	0.93 (0.86;0.97)	0.93 (0.86;0.97)	85.7 (72.8;94.1)	91.4 (81.0;97.1)	9.94	0.16	0.79 (0.70;0.86)

^*^Cutoff points for AST and GGT are 40 U/L

^**^Cutoff point for CDT – “all” group is 1.0%, “men” group is 1.0%, and “women” group is 0.8%

^***^Cutoff point for GGT–CDT – “all” group is 2.1114, “men” group is 3.1901, and “women” group is 1.9765

^****^Cutoff point for AUDIT-C – “all” group is score 4, “men” group is score 5, and “women” group is score 4. (The cutoff point was determined by ROC analyses in the sample)

^*****^At risky drink (men > 14 drinks/week, women > 7 drinks/week)/reference population (men ≦14 drinks/week, women ≦7 drinks/week)

AST = aspartic acid transaminase; AUC = area under the curve; AUDIT-C = Alcohol Use Disorder Identification Test Consumption alcohol use disorder identification test consumption; CDT = carbohydrate-deficient transferrin; GGT = gamma- glutamyltransferase; LR = likelihood ratios; SEN = sensitivity; SPC = specificity

## Discussion

In this study, we investigated the effectiveness of AUDIT-C and biochemical markers in identifying alcohol problems among suicide attempters visiting ED. We found that AUDIT-C is a more powerful tool to screen for alcohol problems among suicide attempters than biochemical markers regardless of sex.

The study results highlight the clinical utility and practical importance of a screening questionnaire to detect alcohol problems in an ED setting. Neumann et al. reported that the screening properties of AUDIT are superior to those of biochemical markers such as %CDT, MCV, and GGT for the detection of alcohol problems among trauma patients, and they are not significantly clinically enhanced by the use of biochemical markers [19]. Other previous studies conducted in primary care and ED settings reported that the AUC of AUDIT-C for detecting any alcohol use disorder was > 0.9 for both men and women, which was comparable with the AUC calculated in our study [19, 27, 42, 43]. Consistent with previous findings, our results confirm that AUDIT-C is a useful and an accurate tool to screen for alcohol problems in various clinical settings, and we suggest that AUDIT-C is a reliable tool to detect alcohol problems among suicide attempters.

We could not confirm which questionnaire is optimal for detecting alcohol problems among suicide attempters because we only used AUDIT-C in this study. According to a recent survey conducted in the UK, although 61.4% of EDs use a formal screening tool for evaluating alcohol problems among adults and the most common tool is AUDIT-C, our study suggests that each hospital should select their optimal screening tool [44]. Further research may be needed to evaluate the optimal alcohol screening tool for suicide attempters.

The limitations of the use of questionnaires arise from the need for patients to cooperate and divulge the information required, thereby lacking objectivity. Furthermore, questionnaires involve the assessment of alcohol problems during a longer prehospitalization period; thus, they potentially provide false-positive results for a recent episode of heavy drinking. The inability of some patients to participate in self-assessment questionnaires due to the severity and acuity of their disease at admission also adds to these limitation [21]. In addition, because there is no established and reliable cutoff points for alcohol use disorder among suicide attempters, cutoff points were determined using ROC analysis, and this may have affected the sensitivity and specificity of the test.

The insufficient accuracy of biochemical markers such as GGT and %CDT among suicide attempters is similar to that observed in earlier studies [45]. In a previous study on patients who visited an ED, [46] CDT and GGT levels showed high sensitivity but low specificity in determining alcoholism, which are similar to the results observed for men in our study. The performances of alcohol-related biochemical markers to detect alcohol problems in the present study were better than those observed in previous studies with AUC of > 0.8 for both men and women. In cases wherein the screening for alcohol problems using AUDIT-C was limited as mentioned above, the use of biochemical markers could be an alternative method. In particular, the use of CDT or GGT–CDT may be recommended considering the results of the present and previous studies [35, 36, 40].

Considering that the alcohol use pattern at the initial suicide attempt appears to be a strong long-term and short-term risk factor for suicide reattempt, [47] screening for the alcohol use pattern among suicide attempters is essential to prevent future suicide attempts, and it will help set treatment plans. Intensive psychiatric treatment or intervention for the risk of future suicide reattempt is possible through the early identification of alcohol problems in the ED. Although the effect of alcohol use on suicide reattempts among suicide attempters is a well-known risk factor for suicide reattempt, studies on whether screening for alcohol use disorder or screening and intervention for it in an ED setting are effective for reducing suicide reattempts or alcohol use disorder among suicide attempters have not been conducted. The results of the studies on screening and brief intervention program related to alcohol use disorder among ED visitors are mixed and not conclusive [48–50]. Thus, further research is needed on the development and validation of intervention programs on alcohol use disorder for suicide attempters.

The findings of our study should be considered in light of some limitations. First, we included subjects who cooperated for an interview and allowed blood collection among the suicide attempters who visited our ED. The subjects likely had more alcohol drinking problems than those who did not participate, which may cause selection bias. Alcohol use disorder was found only in 7–37% of suicide attempters in previous studies, but we found a high incidence of alcohol use disorder, i.e., 60.4% of men and 28.0% of women [51, 52]. The prevalence of disease influences the positive predictive value and negative predictive value of the diagnostic tool but not its sensitivity, specificity, AUC, and predictive accuracy. Therefore, the AUCs of AUDIT-C and biochemical markers were not possibly influenced by the high prevalence of alcohol use disorder in this study, although this limitation should be considered in the interpretation of the results. Second, the study conclusions were derived based on a certain population of suicide attempters, and the most common method of suicide attempt was drug intoxication. This may have affected biochemical markers associated with liver functions such as AST. Third, the study was conducted using a relatively small sample size; it may be necessary to repeat this study using a larger number of subjects. Lastly, we did not use a structured interview to diagnose alcohol use disorder, although we tried to validate the diagnosis with the consensus of two board-certified psychiatrists.

## Conclusions

AUDIT-C presented the highest values of AUC, sensitivity, and specificity for screening for alcohol use disorder and risky drinking than any other biochemical markers in both men and women. In conclusion, we suggest that AUDIT-C is helpful in the assessment of alcohol use disorder among suicide attempters. Early identification of at-risk patients during their hospital course in a more noninvasive manner may allow clinicians to intervene carefully and increase compliance of suicide attempters. Therefore, a careful history taking of alcohol consumption should be performed with subjects or their guardians when a suicide attempter presents to an ED.

## Data Availability

The datasets used and analyzed during the current study are available from the corresponding author on reasonable request.

## Abbreviations

AST: Aspartate aminotransferase
AUDIT-C: Alcohol Use Disorder Identification Test Consumption
CDT: Carbohydrate-deficient transferrin
DSM-IV-TR: Diagnostic and statistical manual of mental disorders-IV-Text Revision
ED: Emergency department
GGT: Gamma-glutamyltransferase
